# Successful resection of a cavernous hemangioma involving the rectal muscularis propria layer by endoscopic full-thickness resection

**DOI:** 10.1055/a-2081-9202

**Published:** 2023-05-26

**Authors:** Wei Liu, Yinong Zhu, Xianglei Yuan, Bing Hu

**Affiliations:** Department of Gastroenterology and Hepatology, West China Hospital, Sichuan University, Chengdu, P. R. China

A 46-year-old woman with a history of hematochezia visited our institution for colonoscopy. A globular submucosal tumor with a diameter of approximately 20 mm and mucosal hyperemia was detected in the rectum ( Fig. 1 a ). Further endoscopic ultrasonography showed a well-defined, homogeneous, hyperechoic mass 18 mm × 9 mm in size originating from the submucosal layer ( Fig. 1 b ), suggesting that the mass might be a hemangioma. At this point, endoscopic submucosal dissection (ESD) was considered for the treatment of the lesion. First, after submucosal injection we made a circumferential mucosal incision using a dual knife (Olympus, Tokyo, Japan). However, during the procedure we found that the boundary between the lesion and the muscularis propria layer was not clear ( Fig. 1 c ). Therefore, ESD was not the right choice to ensure en bloc resection of the lesion, and endoscopic full-thickness resection (EFTR) was believed to be a better option for this patient ( Fig. 1 d , Fig. 2 a , Video 1 ). After partial dissection of the lesion, we used a snare to complete EFTR of the lesion, and finally the defect was successfully closed using titanium clips and a nylon cord (Micro-Tech, Nanjing, China) ( Fig. 1 e ). Histopathology revealed a cavernous hemangioma with involvement of the muscularis propria layer ( Fig. 2 b ). The patient was discharged 3 days after treatment without any complications. A follow-up colonoscopy was performed 3 months later and indicated that the defect was basically healed ( Fig. 1 f ).

**Fig. 1:**
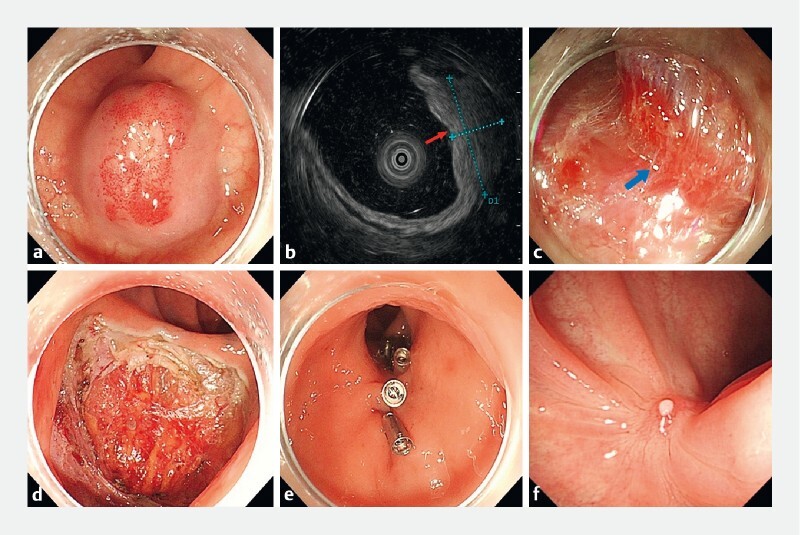
Successful en bloc resection of rectal cavernous hemangioma by endoscopic full-thickness resection. **a** Colonoscopy revealed a submucosal tumor approximately 20 mm in diameter in the rectum. **b** Endoscopic ultrasonography showed a well-defined, homogeneous, hyperechoic mass 18 mm × 9 mm in size growing from the submucosal layer (red arrow). **c** During treatment it became evident that the lesion had involved the muscularis propria layer (blue arrow). **d** The lesion was successfully resected by endoscopic full-thickness resection. **e** The postoperative defect was closed. **f** Follow-up colonoscopy 3 months later showed that the defect was basically healed.

**Fig. 2:**
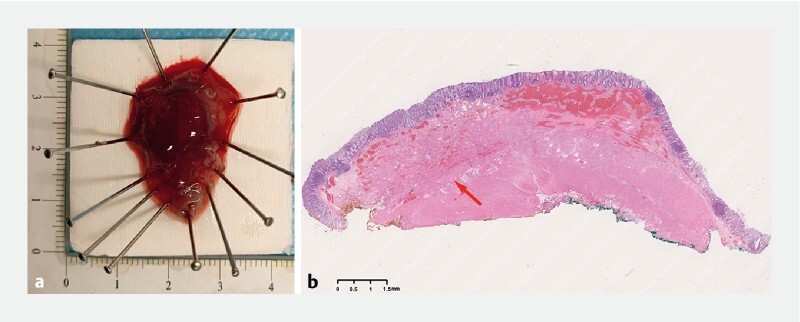
Postoperative specimen and histopathological result. **a** The specimen measured 30 mm × 20 mm. **b** Histopathology revealed a cavernous hemangioma involving the muscularis propria layer (red arrow).

**Video 1** Successful resection of a cavernous hemangioma involving the rectal muscularis propria layer by endoscopic full-thickness resection.


Although endoscopic mucosal resection and ESD have been reported for treatment of colorectal cavernous hemangioma 1 2 , this is the first report of a cavernous hemangioma resected by EFTR. Since hemangiomas sometimes infiltrate into the muscle layer or completely over the layer 3 , compared with endoscopic mucosal resection and ESD, the major advantage of EFTR is that it carries less risk of residual or recurrent hemangioma, and it is suggested that perhaps EFTR is a better treatment option for colorectal cavernous hemangiomas involving the muscularis propria layer.

Endoscopy_UCTN_Code_TTT_1AQ_2AD

## Notes

Endoscopy E-Videos

https://eref.thieme.de/e-videos
*E-Videos* is an open access online section of the journal *Endoscopy* , reporting on interesting cases and new techniques in gastroenterological endoscopy. All papers include a high-quality video and are published with a Creative Commons CC-BY license. Endoscopy E-Videos qualify for HINARI discounts and waivers and eligibility is automatically checked during the submission process. We grant 100% waivers to articles whose corresponding authors are based in Group A countries and 50% waivers to those who are based in Group B countries as classified by Research4Life (see: https://www.research4life.org/access/eligibility/ ). This section has its own submission website at https://mc.manuscriptcentral.com/e-videos
